# Can preoperative results predict the need for future reintervention following injection laryngoplasty for unilateral vocal fold paralysis?

**DOI:** 10.1007/s00405-021-06925-z

**Published:** 2021-06-09

**Authors:** Beata Miaśkiewicz, Aleksandra Panasiewicz, Elżbieta Gos, Paulina Krasnodębska, Piotr H. Skarżyński, Agata Szkiełkowska

**Affiliations:** 1 Audiology and Phoniatric Clinic, World Hearing Centre, Institute of Physiology and Pathology of Hearing, Mokra 17 Str., Kajetany, Nadarzyn, 05-830 Warsaw, Poland; 2 Department of Teleaudiology and Screening, World Hearing Center, Institute of Physiology and Pathology of Hearing, Mokra 17 Str., Kajetany, Nadarzyn, 05-830 Warsaw, Poland

**Keywords:** Unilateral vocal fold paralysis, Injection laryngoplasty, Calcium hydroxylapatite, Hyaluronic acid, Perceptual voice evaluation, Acoustic voice assessment

## Abstract

**Purpose:**

The objective was to investigate whether a patient’s preoperative test results can predict the need for future reoperation in unilateral vocal fold paralysis (UVFP).

**Methods:**

A single-centre retrospective study was performed. The study group consisted of 18 patients with UVFP who had been treated with injection laryngoplasty but who required further treatment and were augmentated again within 36 months. The control group consisted of 33 injected patients who had not required reintervention up to 36 months later.

**Results:**

Only glottal gap was associated with a relative risk for reinjection. Glottal gap was found to be severe in 77.8% of the patients from the study group compared to 42.4% of the controls, and the difference was statistically significant. The kind of injected material (calcium hydroxylapatite or hyaluronic acid), age, and voice assessment (perceptual, objective, or subjective) did not seem to affect the likelihood of reoperation being needed. There were no between-group statistically significant differences in individual aspects of the GRBAS scale. The global score was slightly higher in the study group, but it did not reach statistical significance (*U* = 198.5; *p* = 0.09). A comparison of VHI scores did not yield statistically significant differences between the study and control groups. No significant differences in objective acoustic voice parameters were observed between the groups.

**Conclusion:**

Only glottal gap occurred to be associated with a relative risk for reinjection. A kind of injected material (CaHA or HA), age, perceptual, objective and subjective voice assessment do not seem to impact the likelihood of reoperation in patients with UVFP.

## Introduction

Unilateral vocal fold paralysis (UVFP) occurs in approximately 0.41–0.51% of the population, with voice deterioration present in 83.6% of cases [1]. UVFP results from unilateral injury of the vagus nerve or the recurrent laryngeal nerve, mostly due to surgery (47–56%) or idiopathically (12–37%) [2]. The consequences of UVFP are significant functional deficits manifesting as dysphonia, with a breathy, weak voice resulting from glottal insufficiency, dysphagia, or aspiration.

Treatment for UVFP not only focuses mainly on restoration of the voice by decreasing the glottal gap and recovering a mucosal wave, but also includes voice therapy and surgery.

A wide variety of surgical interventions are currently available for treatment of nonresolving UVFP, including injection laryngoplasty (IL), medialization thyroplasty, arytenoid adduction, and reinnervation.

Injection laryngoplasty, introduced by Brunings in 1911, is a minimally invasive technique that, over decades, has proven its effectiveness in restoring the voice in UVFP. The vocal fold injection techniques can be divided into temporary or more permanent procedures, depending on the augmented material.

A number of factors seem to influence the clinical effectiveness and durability of IL treatment, including the kind of injectable material (temporary or more permanent), extent of glottal gap, points of injection, onset of injury, cause, prognosis, age, and comorbidities. In clinical practice, some patients with UVFP who are initially treated with IL require further reintervention some months to several years later. It would be useful to predict the degree of voice benefits after IL in order to select an optimal treatment plan**.**

The objective was to investigate whether a patient’s preoperative test results can predict the need for the future reoperation in unilateral vocal fold paralysis (UVFP).

## Material and methods

A single-centre retrospective study was performed from June 2010 to March 2018 at the Institute of Physiology and Pathology of Hearing, Kajetany/Warsaw, Poland. There were two groups of the patients. The study group consisted of 18 patients with UVFP treated with injection laryngoplasty, but who required further treatment and reinjection within 36 months of the initial operation. The control group consisted of 33 augmented patients who did not require reintervention within 36 months.

The inclusion criteria were as follows: patients over 18 years old, having glottal insufficiency and/or dysphonia due to UVFP, having had an IL procedure during which the paralysed vocal fold was injected with either calcium hydroxylapatite (CaHA) and/or hyaluronic acid (HA), and available to fill out a voice quality questionnaire (the Voice Handicap Index). According to the literature, the average time of benefits from CaHA augmentation is about 36 months, and we took this time frame as the criterion for selecting patients for the study and control groups [3]. The following were the exclusion criteria: history of prior laryngeal surgery, follow-up after IL shorter than 36 months, and inability to fill out the voice quality questionnaire.

IL was performed under general anaesthesia using suspended microlaryngoscopy and endotracheal intubation. All operations were performed by the senior author (BM). We used the following two injectable materials: hyaluronic acid (Surgiderm 24 XP, Allergan) and calcium hydroxylapatite (Radiesse Voice Implant, Merz Aesthetics). From June 2010 to January 2015 IL was performed using HA only; from January 2015 both HA and CaHA were used.

Augmentation was performed with a 25 gauge (0.5 mm) laryngeal needle. Because of the different properties of each substance, the areas of injection were different for each material. HA was injected as close as possible to the deep layer of the lamina propria, until the volume of the vocal fold was mildly overcorrected. The main injection was made anteriorly to the vocal process; if necessary, further ones were placed laterally and medially. The mean quantity of HA used was 0.76 mL in the study group and 0.96 mL in the control group. In the case of CaHA, the most common injection approach involved application at one or two points laterally to the superior arquate line at the posterior third and/or mid-membranous vocal fold. As recommended for CaHA, augmentation was performed to 10–15% overcorrection [4]. The mean amount of CaHA injected was 1.5 mL in the study group and 0.91 mL in the control group.

The Voice Handicap Index questionnaire (VHI-30) was administered to evaluate each patient’s perception of their own voice [5]. The VHI-30 total score (VHI-T) and its components—emotional (VHI-E), physical (VHI-P), and functional (VHI-F) subscale scores—were all calculated.

Laryngovideostroboscopy (LVS) was performed with a 70° rigid laryngoscope (EndoStrob DX Xion 327, GmbH, Germany), and glottal closure was assessed subjectively. The pattern of glottal closure was rated on a 5-point scale according to the proposal of Lundy et al. [6] as follows: 0—none, no appreciable gap; 1—minimal, a minimal posterior gap involving the nonmembranous portion of the folds; 2—small, a small gap extending up to one-third of the posterior membranous vocal folds; 3—moderate, a moderate gap extending up to two-thirds of the posterior membranous vocal folds; 4—severe, a severe gap where there was no observable contact between the vocal folds. All stroboscopic videos were evaluated preoperatively, and then the recordings were retrospectively assessed anonymously by the senior author, a laryngologist/phoniatrist.

An auditory-perceptual evaluation of the patients’ voices was carried out with the use of the GRBAS scale [7] in which a clinician estimates the grade of hoarseness (G), roughness (R), breathiness (B), asthenia (A), and strain in the voice (S) on a scale from 0 to 3 (0, normal; 1, mild; 2, moderate; 3, severe). Ratings based on the patient’s sustained phonation and a short speech sample were made by the senior author upon each clinic presentation. Then, the retrospectively performed blinded evaluation of the recorded voice samples was carried out by the same researcher.

An objective acoustic voice analysis was performed with a Computerized Speech Lab (CSL) 4500 external module from Kay Elemetrics Corporation (Lincoln Park NJ). All voices were recorded with an ECM 800 microphone (Behringer) positioned approximately 15 cm from the mouth at an angle of 45° to reduce airflow effects. Analysis of a voice sample recorded at a sample rate of 25 kHz was done using the Multidimensional Voice Program software (MDVP 5105 version 2.7.0). Three samples of the sustained vowel “a” in modal voice were used for analysis; only the middle portion of the uttered vowel was used (min. 0.6 s), avoiding onset and offset effects [8]. The following acoustic parameters were calculated: average fundamental frequency (F0), frequency variations (% Jitter; Relative Average Perturbation, RAP; Pitch Perturbation Quotient, PPQ; Smoothed Pitch Perturbation Quotient, sPPQ; Fundamental Frequency Coefficient Variation, vF0), amplitude variations (% Shimmer; Amplitude Perturbation Quotient, APQ; Smoothed Amplitude Perturbation Quotient, sAPQ; Peak-to-Peak Amplitude Coefficient of Variation, vAm), and noise-related parameters (Noise to Harmonic Ratio, NHR; Soft Phonation Index, SPI).

### Statistical analysis

Test outcomes obtained before IL were compared in the study and control groups. A Mann–Whitney *U*-test and a chi-square test were used to test differences between the groups. Then, multivariate analysis was performed. Logistic regression analysis was used for prediction of group membership. Only variables with *p* < 0.2 in the previous univariate analyses were included in the model. Statistical significance was specified as a *p*-value less than 0.05. The analysis was performed using IBM SPSS Statistics, version 24.

## Results

Patient characteristics are presented in Table 1.

**Table 1:** Characteristics of the study and control groups

		Study group (*n* = 18)	Control group (*n* = 33)	Test result
Sex	Women	10 (55.6)	22 (66.7)	*χ*^2^ = 0.62; *p* = 0.433
	Men	8 (44.4)	11 (33.3)	
Age	Range	40–85	29–78	*U* = 234.5; *p* = 0.218
	M (SD)	60.5 (12.4)	55.3 (13.5)	
Duration of paralysis (years)	Range	0.5–23	0.5–33	*U* = 231.5; *p* = 0.191
	M (SD)	4.3 (6.3)	3.9 (5.8)	
Paralysed VF	Right	7 (38.9)	13 (39.4)	*χ*^2^ = 0.01; *p* = 0.972
	Left	11 (61.1)	20 (60.6)	
Augmented VF	Right	4 (22.2)	11 (33.3)	*χ*^2^ = 0.71; *p* = 0.702
	Left	8 (44.4)	13 (39.4)	
	Both	6 (33.4)	9 (27.3)	
Injected material (to a paralysed VF)	CaHA	7 (38.9)	8 (24.2)	*χ*^2^ = 1.23; *p* = 0.541
	HA	9 (50.0)	21 (63.6)	
	CaHA & HA	2 (11.1)	4 (12.1)	
Amount of injected material (in all)	Range	0.4–1.8	0.94 (0.36)	*U* = 2 50.5; *p* = 0.897
	M (SD)	0.4–2.1	0.97 (0.41)	
Rehabilitation before augmentation	Yes	14 (77.8)	24 (72.7)	*χ*^2^ = 0.16; *p* = 0.692
	No	4 (22.2)	9 (27.3)	
Rehabilitation after augmentation	Yes	9 (50.0)	19 (57.6)	*χ*^2^ = 0.27; *p* = 0.603
	No	9 (50.0)	14 (42.4)	

*M* mean, *SD* standard deviation, *U* result of Mann–Whitney test, *p* *p*-value, *χ*^2^ result of chi-square statistic test

As can be seen in Table 1, the patients from the study group were slightly older than the controls and the duration of paralysis was somewhat longer; however, the differences did not reach statistical significance. Generally, we did not observe any statistically significant differences between the study and control groups, either in sociodemographic characteristics or in variables concerning glottal insufficiency.

The etiology of UVFP was diverse and these data are summarized in Table 2.

**Table 2:** Etiology of unilateral vocal fold paralysis

	Study group (*n* = 18)	Control group (*n* = 33)
Thyroidectomy	10 (55.6)	19 (57.6)
Idiopathic	5 (27.8)	7 (21.2)
Intracranial tumor	1 (5.6)	4 (12.2)
Mediastinal surgery	1 (5.6)	1 (3.0)
Spine surgery	–	1 (3.0)
Postintubation	–	1 (3.0)
Radiotherapy for carcinoma of palatine tonsil	1 (5.6)	–

Percentages are given in brackets

### Glottal gap assessment

Glottal gap was assessed as small, moderate, or severe in all patients and the results before IL are shown in Table 3.

**Table 3:** Glottal gap assessment

Glottal gap	Study group (*n* = 18)	Control group (*n* = 33)	Test result
Small	1 (5.6)	8 (24.2)	*χ*^2^ = 6.14; *p* = 0.047
Moderate	3 (16.8)	11 (33.3)	
Severe	14 (77.8)	14 (42.4)	

*χ*^2^ result of chi-square statistic test, *p p*-value

Glottal gap was found to be severe in the majority of the patients from the study group (77.8%) and in 42.4% of the controls. Small glottal gap was revealed only in one patient (5.6%) from the study group and in 24.2% of the controls. Taken together, glottal gap was larger in the study group than in the control group and the difference was statistically significant.

### Perceptual evaluation (GRBAS)

Data on GRBAS parameters are presented in Table 4.

**Table 4:** GRBAS parameters in the study and control groups

	Study group (*n* = 17)				Control group (*n* = 33)				*χ*^2^; *p*
	0	1	2	3	0	1	2	3	
G	–	3 (17.6)	12 (70.6)	2 (1.8)	1 (3.0)	13 (39.4)	17 (51.5)	2 (6.1)	3.33; 0.343
R	–	6 (35.3)	8 (47.1)	3 (17.6)	–	12 (36.4)	18 (54.5)	3 (9.1)	0.81; 0.667
B	–	2 (11.8)	12 (70.6)	3 (17.6)	2 (6.1)	10 (30.3)	16 (48.5)	5 (15.2)	3.66; 0.301
A	–	5 (29.4)	12 (70.6)	–	5 (15.2)	12 (36.4)	12 (36.4)	4 (12.1)	7.53; 0.057
S	–	8 (47.1)	9 (52.9)	–	2 (6.1)	19 (57.6)	11 (33.3)	1 (3.0)	2.85; 0.415

*G* grade, *R* roughness, *B* breathiness, *A* asthenia, *S* strain, *0* normal, *1* mild, *2* moderate, *3* severe, *χ*^2^ result of chi-square statistic test, *p p*-value

Percentages are given in brackets

There were no patients in the study group whose voice was assessed as normal in GRBAS, whereas in the control group there were patients who had normal results in grade, breathiness, asthenia, and strain. There were no between-group statistically significant differences in individual aspects of GRBAS. The global score (a sum of the five parameters) was slightly higher in the study group (*M* = 9.06; SD = 2.19) than in the controls (*M* = 7.85; SD = 2.87), but it did not reach statistical significance (*U* = 198.5; *p* = 0.09).

### Voice Handicap Index results

**Fig. 1:**
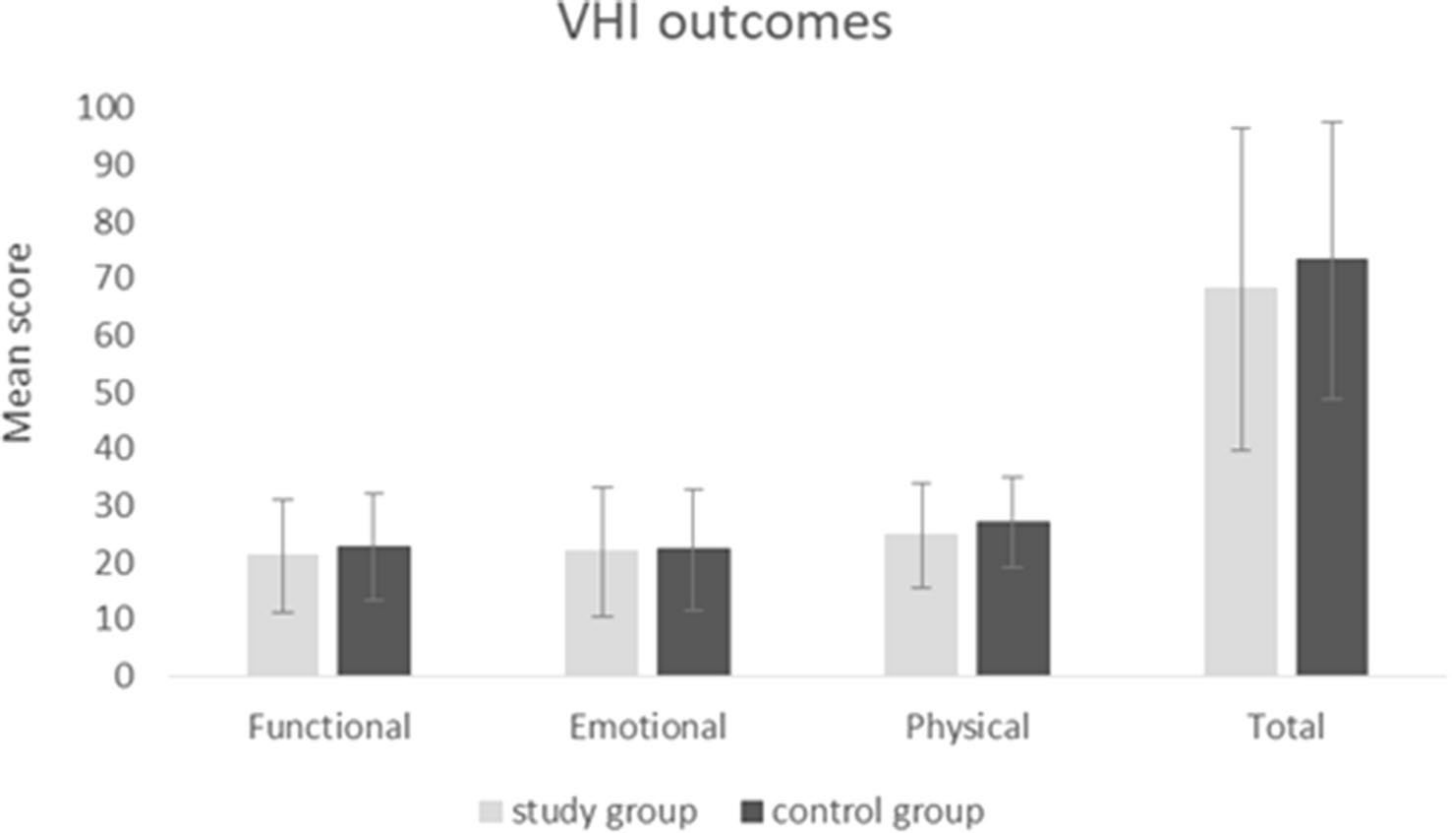
Voice Handicap Index scores in the study and control groups. Error bars represent standard deviations

Figure 1 shows the preoperative subscales scores (functional, physical, and emotional) and total scale score of VHI in both groups. The VHI outcomes were similar in the patients who needed reintervention and in patients who did not need reintervention. Comparison of the VHI scores did not yield a statistically significant difference between the study and control groups.

### Acoustic assessment

The comparison of the voice parameters in the study group with those in the control group is shown in Table 5.

**Table 5:** MDVP parameters in the study and control groups

	Study group (*n* = 17)		Control group (*n* = 32)		*U*	*p*
	M	SD	M	SD		
Fo	194.29	72.82	183.72	67.38	266.0	0.900
Jitt	5.57	6.17	6.92	5.79	205.0	0.900
RAP	3.23	3.60	3.99	3.31	202.0	0.159
PPQ	3.52	3.79	3.96	3.03	220.0	0.141
sPPQ	5.74	6.94	4.76	4.19	176.0	0.272
vFo	9.59	10.22	8.06	6.06	256.0	0.615
Shim	12.17	7.64	11.52	4.16	249.0	0.737
APQ	8.72	5.63	8.2	3.22	232.0	0.755
sAPQ	10.39	5.76	10.61	5.49	197.0	0.743
vAm	23.38	9.76	19.55	7.41	222.0	0.294
NHR	0.32	0.25	0.66	1.24	190.0	0.149
SPI	9.93	6.33	9.44	5.97	256.0	0.737

*M* mean, *SD* standard deviation, *U* result of Mann–Whitney test, *p* *p*-value

No significant differences in the voice parameters were observed between the groups.

### Multivariate analysis

Logistic regression analysis was used for prediction of group membership. Six variables were included in the model (duration of paralysis, glottal gap, GRBAS A, RAP, PPQ, and NHR). Results are shown in Table 6.

**Table 6:** Multivariate analysis for prediction of a group membership based on preoperative results

	*β*	Wald statistic	*p*-value	OR
Duration of paralysis	0.029	0.300	0.584	1.030
Glottal gap	1.471	4.506	0.034	4.355
GRBAS A	0.077	0.016	0.901	1.081
RAP	0.974	1.949	0.163	2.648
PPQ	− 1.174	2.612	0.106	0.309
NHR	2.687	1.607	0.205	14.682
Constant	− 6.225	6.702	0.010	0.002

*β *logistic regression coefficient, *OR *odds ratio

Only one variable was found to be a statistically significant predictor. On the basis of glottal gap assessment it was possible to predict the preoperative period to which each patient belonged. The more severe the glottal gap, the higher the probability that a patient would need reintervention on their vocal folds.

## Discussion

Treatment of UVFP aims to reduce glottal insufficiency and improve voice quality through surgical procedures, voice therapy, or a combination of the two. Effective management requires accurate diagnostic and prognostic information to select an individual treatment plan.

In this study, we evaluated different factors that could impact the need for reoperation in patients with UVFP who had previously undergone injection laryngoplasty.

We took the 36-month cut off point for selecting patients for the study and control groups independently on a type of an augmented agent. Even though Ha is commonly known as a short-term lasting material, there are also some studies reporting longer durability of HA than it is generally considered [9, 10].

We found a statistically significant relationship between the preoperative glottal gap and the need for reintervention after IL in UVFP. Glottal insufficiency was larger in patients who required reaugmentation within 36 months after the first injection than in individuals who did not require reinjection.

Some authors suggest that the critical preoperative glottal gap is 2 mm, and if the gap is larger than this satisfactory long-term effects after injection augmentation cannot be expected [9, 11]. There are also some reports of augmentation in cases of severe (up to 3 mm) glottal insufficiency, but satisfactory effects were shorter and patients usually required surgical reintervention [9, 12]. Fang et al. reported on 42 patients with UVFP who underwent conservative treatment (*n* = 22) or early injection laryngoplasty with HA (*n* = 20); patients with an initially large glottal gap had a higher incidence of further permanent laryngoplasty [13]. Reiter et al. evaluated voice outcomes in 19 UVFP patients treated with HA injection; at 12 months’ follow-up they found that 42% of patients required further surgical treatment and the mean glottal gap was 2.8 mm [9]. Rosen et al. reported that, after CaHA augmentation, 13% of their UVFP patients required further surgical treatment at the 12-month follow-up. They considered that initial underinjection, progression of vocal fold paresis, or loss of injection agent were the causes of reintervention [14]. There are also two studies which report that the treatment decisions in UVFP may not always be predicated on the size of the glottal gap alone [12, 15].

Voice deterioration in UVFP has an impact on acoustic parameters and perceptual evaluation. The lack of complete closure creates a puff of air which affects vibratory amplitude. [8]. Several studies have shown a consistent increase in pitch and amplitude in UVFP and a positive correlation to the degrees of roughness and breathiness [16, 17]. In our study we observed abnormal values of all acoustic parameters, but the differences between groups were not statistically significant. The pitch perturbation variables were not found to be predictors for reintervention after IL. Although the A parameter of the GRBAS scale achieved a difference quite close to statistical significance, it was not predictive of reintervention.

After the onset of paralysis, patients from both groups were injected an average of 4.3 years (the study group) or 3.9 years (the control group) later, with the minimum period 6 months. We did not observe a statistically significant difference between the groups, and so this variable is not highly predictive of reintervention after IL. Recently, a number of studies have recommended early injection laryngoplasty for symptoms of glottal insufficiency. The authors reported lower rates of permanent procedures being necessary in the future, even when the immobility lasts beyond the expected spontaneous neural recovery period [18]. These works suggest that early augmentation allows for a well-positioned vocal fold and promotes more favourable reinnervation [18, 19]. These reports lead to the hypothesis that the longer the period from the onset of injury, the more the progression of vocal fold paresis, resulting in decreased vocal fold volume and tension, increased glottal insufficiency, and consequently a higher likelihood of the need for reintervention.

The type of material injected is one factor that has been considered important in determining how long IL effects last. *Several studies suggest that hyaluronan-based biomaterials may be better suited for injection into the lamina propria,* while CaHA has been recommended for injection into the paraglottal space [14, 20–23]. Our results did not show any statistically significant difference between the groups in terms of the augmented material used. Reiter et al. reported a 42% reintervention rate (mostly thyroplasty) in UVFP because of loss of HA 6 months after initial augmentation [9]. Hertegard et al. observed a 25% reinjection rate at 1- to 2-year follow-up because of partial resorption of HA [10]. As for application of CaHA in patients with UVFP, the rate of those needing further surgical treatment after CaHA injection was reported as 13% at 1-year follow-up [14].

Some authors suggest that underaugmentation of the vocal fold may cause rapid loss of benefits at 4 months or less, after which reinjection is required [3, 14]. In the present study the average amounts of injected substances did not differ statistically between the groups.

Progression of vocal fold atrophy, both paralyzed and nonparalyzed, resulting from age should also be mentioned [1, 22]. Although one might expect that older patients would have a higher chance of needing reintervention after IL—due to age-related functional, structural, and biochemical changes—we did not find a statistically significant difference between the groups in terms of age. Similar results have been reported by Mor et al. concerning predictors for permanent medialization laryngoplasty in UVFP [19].

Although dysphonia is often emphasized as a reason for intervention in UVFP, subjective voice assessment as measured by VHI-30 score did not correlate with the need for reaugmentation. We did not find a statistically significant difference between groups in terms of any VHI-30 subscale or total score. Similarly, Mor et al. did not find any correlation of VHI-10 score with the likelihood of permanent medialization laryngoplasty in UVFP [19].

The use of voice therapy during the preoperative or postoperative period aims to maximize vocal efficiency, modify aberrant vocal habits like supraglottal hyperfunction or respiratory irregularity, and facilitate compensation. Voice therapy also delays the onset of atrophy in paralysed muscle [24]. In this study we did not find any statistically significant difference between groups concerning the use of voice therapy (preoperative or postoperative).

Our study has some limitations. The patient population was relatively small. Because the present paper is based on a retrospective study some patients dropped out of the study or some data were not available in all periods. We would like to see a prospective study with a larger cohort. We consider the subjective assessment of glottal gap as a limitation of our study. Two studies have described methods for objectively calculating the glottal gap size [13, 15], but they have not been widely used. However, others consider visual judgement based on laryngovideostroboscopy to be a good diagnostic test and have used it as their gold standard [25, 26].

## Conclusions

In this study we have analysed some factors that might be predictive of the need for reintervention after initial IL in patients with UVFP. Only glottal gap turned out to be associated with increased risk for reaugmentation. The more severe the glottal gap, the higher the probability that a patient will need reintervention on their vocal folds.

The kind of injected material (CaHA or HA), age, and voice assessment (perceptual, objective, or subjective) does not seem to affect the likelihood of reoperation in patients with UVFP.
